# Engineering Classical Capacity of Generalized Pauli Channels with Admissible Memory Kernels

**DOI:** 10.3390/e23111382

**Published:** 2021-10-21

**Authors:** Katarzyna Siudzińska, Arpan Das, Anindita Bera

**Affiliations:** Institute of Physics, Faculty of Physics, Astronomy and Informatics, Nicolaus Copernicus University in Toruń, ul. Grudziądzka 5, 87-100 Toruń, Poland; arpand@umk.pl (A.D.); anindita.bera@umk.pl (A.B.)

**Keywords:** classical capacity, generalized Pauli channels, non-Markovian evolution, memory kernels

## Abstract

In this paper, we analyze the classical capacity of the generalized Pauli channels generated via memory kernel master equations. For suitable engineering of the kernel parameters, evolution with non-local noise effects can produce dynamical maps with a higher capacity than a purely Markovian evolution. We provide instructive examples for qubit and qutrit evolution. Interestingly, similar behavior is not observed when analyzing time-local master equations.

## 1. Introduction

In quantum information processing, it is crucial to understand how to transmit, manipulate, and preserve quantum information sent through a noisy quantum channel [1,2]. Due to scientific and technological advancements, logic gates and other electronic devices are approaching atomic scales. Therefore, it is becoming increasingly hard to reliably transfer information. This can be remedied if one can minimize the detrimental effects of noise through error correction, error mitigation, or error suppression techniques [3,4].

However, removing errors is only one way to deal with undesirable effects of environmental noise on quantum systems. Another approach to the problem is, instead of reducing the noise, using it to one’s advantage. This perception of the role of environmental noise was popularized by the observation that dissipation can be used to enhance quantum information processing [5]. In this way, dissipation has become a quantum resource that is exploited to manipulate quantum systems and engineer specific properties of quantum channels [6,7,8]. In particular, the memory effects caused by environmental noise have been used for performing quantum information processing tasks, such as improving channel fidelity or preserving quantum entanglement [9]. A decrease in error accumulation was achieved for dissipative Markovian processes and their generalizations [10,11], where adding noises to the Markovian evolution slows down the rate at which the system approaches a steady state.

The goal of this paper is to show how to engineer quantum noise to improve the channel capacity, which is a very important measure in quantum computation and quantum information theory. Through the channel capacity, one can determine the amount of information transmitted coherently through a quantum channel. However, in contrast to the classical channels, which have a unique (Shannon) capacity, the concept of quantum channel capacity is more complex, giving rise to a whole range of informational characteristics. If quantum information is transferred through a noisy channel, then one must consider the quantum capacity, whose lower and upper estimations were determined by Lloyd [12], Shor [13], and Devetak [14]. In quantum cryptography, communication tasks often require the use of private classical capacity [14]. Additionally, quantum correlations are essential to the entanglement-assisted capacity [15], which is the highest rate of classical information transition. The problem of simultaneously transferring classical and quantum information was investigated by Devetak and Shor [16]. More information about channel capacities is available in review works, see e.g., [17,18].

The capacity that directly generalizes the notion of Shannon capacity for classical channels to the quantum scenario is classical capacity [19,20]. In this case, classical information is sent through a quantum channel using separable input states and joint measurements of the outputs. Recently, there has been significant interest in calculating the classical capacity of quantum channels. Rehman et al. used the majorization procedure to provide lower and upper estimations of the Holevo capacity of the Weyl channels [21,22]. Amosov calculated the classical capacity for deformations of classical-quantum Weyl channels [23] and channels generated by irreducible projective unitary representations of finite groups [24].

In this paper, we analyze the time evolution of the classical capacity for the generalized Pauli channels [25,26]. In particular, we compare the capacity for the dynamical maps governed by the memory kernel

(1) K(t)=Lδ(t)+K(t)

with that of the Markovian generator L alone. In the above formula, K(t) is the part of the kernel that does not include the local part with the Dirac delta function δ(t). With the proper choice of parameters, we propose a number of cases where the classical capacity of the map generated by K(t) is better than that of the Markovian semigroup $\Lambda^{M}\left(t\right)=e^{tL}$. Hence, it is shown that non-local memory effects can be effectively used to decrease the error rate of a quantum channel. We also present a class of quantum evolution where the generator L(t) is time-local. This implies that improving the channel capacity is possible not only for the Markovian semigroup but for general Markovian dynamics.

## 2. Generalized Pauli Channels

An important class of quantum channels consists of mixed unitary channels, where a unitary evolution is disrupted by classical errors [27,28]. The channel noise can be corrected with the classical information obtained by measuring the environment [29]. For qubit systems, one considers the Pauli channel [30,31]

(2) $$\Lambda \left[\rho \right]=\sum_{\alpha =0}^{3}p_{\alpha }\sigma_{\alpha }\rho \sigma_{\alpha },$$

where $p_{\alpha }$ is a probability distribution and $\sigma_{0}=I_{2},\sigma_{1},\sigma_{2},\sigma_{3}$ are the Pauli matrices. As the Kraus representation of a quantum map is not unique, it is often more convenient to work with its spectrum. One can find the eigenvalues of the Pauli channel through its eigenvalue equations

(3) $$\Lambda \left[\sigma_{\alpha }\right]=\lambda_{\alpha }\sigma_{\alpha },\;\lambda_{0}=1.$$

An important property of $\sigma_{\alpha }$, where α=1,2,3, is that their eigenvectors $\left\{\psi_{0}^{\left(\alpha \right)},\psi_{1}^{\left(\alpha \right)}\right\}$ form three mutually unbiased bases (MUBs). Recall that two orthonormal bases are mutually unbiased if and only if

(4) $$|\langle \psi_{k}^{\left(\alpha \right)}|\psi_{l}^{\left(\beta \right)}\rangle {|}^{2}=\frac{1}{d}$$

for α≠β and k,l=0,…,d−1, where *d* is the dimension of the underlying Hilbert space (d=2 for qubits).

The Pauli channels can be generalized in multiple ways [32,33,34,35], but only one generalization ensures that the MUB property of its eigenvectors carries over to d>2. Consider the *d*-dimensional Hilbert space H that admits the maximal number of d+1 mutually unbiased bases [36]. Using the rank-1 projectors $P_{k}^{\left(\alpha \right)}=|\psi_{k}^{\left(\alpha \right)}\rangle \langle \psi_{k}^{\left(\alpha \right)}|$, one can define $d^{2}-1$ unitary operators

(5) $$U_{\alpha }^{k}=\sum_{l=0}^{d-1}\omega^{kl}P_{l}^{\left(\alpha \right)},\;\omega =e^{2\pi i/d}.$$

The generalized Pauli channel is constructed as follows [25,26],

(6) $$\Lambda \left[\rho \right]=p_{0}\rho +\frac{1}{d-1}\sum_{\alpha =1}^{d+1}p_{\alpha }\sum_{k=1}^{d-1}U_{\alpha }^{k}\rho U_{\alpha }^{k†},$$

where the Pauli channel in Equation (2) is reproduced after setting d=2. The eigenvalues $\lambda_{\alpha }$ of Λ are real and (d−1)-times degenerated. They satisfy the eigenvalue equations

(7) $$\Lambda \left[U_{\alpha }^{k}\right]=\lambda_{\alpha }U_{\alpha }^{k},\;k=1,\ldots ,d-1,$$

and $\Lambda \left[I_{d}\right]=I_{d}$. In terms of the probability distribution $p_{\alpha }$,

(8) $$\lambda_{\alpha }=\frac{1}{d-1}\left[d(p_{0}+p_{\alpha })-1\right],$$

whereas the inverse relation reads

(9) $$\begin{array}{cc} p_{0} & =\frac{1}{d^{2}}\left(1+\left(d-1\right)\sum_{\alpha =1}^{d+1}\lambda_{\alpha }\right),\\ p_{\alpha } & =\frac{d-1}{d^{2}}\left(1+d\lambda_{\alpha }-\sum_{\beta =1}^{d+1}\lambda_{\beta }\right). \end{array}$$

The complete positivity of the generalized Pauli channel is fully controlled by its eigenvalues. Indeed, Λ is completely positive if and only if $\lambda_{\alpha }$ satisfies the generalized Fujiwara–Algoet conditions [25,37,38]

(10) $$-\frac{1}{d-1}\leq \sum_{\beta =1}^{d+1}\lambda_{\beta }\leq 1+d\min_{\beta >0}\lambda_{\beta }.$$

## 3. Classical Capacity of Generalized Pauli Channels

In the classical theory of information, there exists a unique measure for the amount of information that can be reliably transmitted through a noisy channel. This measure is known as the Shannon capacity, and it is a maximization of the mutual information between the input and output states over all random variable probability distributions [39]. In quantum information theory, however, information can be transmitted in a number of ways. Therefore, there exist many types of channel capacities, such as the quantum capacity [12,13,14], private classical capacity [14], and entanglement-assisted capacity [15]. A direct analogue of the Shannon capacity in the quantum scenario is the Holevo capacity. It determines the maximal amount of classical information that can be reliably transferred, provided that the input state is separable and the output state is measured via joint measurements [17,40]. The Holevo capacity χ(Λ) is defined as the maximal value of an entropic expression [19,20],

(11) $$\chi \left(\Lambda \right)=\max_{\left\{p_{k},\rho_{k}\right\}}\left[S\left(\sum_{k}p_{k}\Lambda \left[\rho_{k}\right]\right)-\sum_{k}p_{k}S\left(\Lambda \left[\rho_{k}\right]\right)\right],$$

where Λ is a quantum channel and S(ρ)=−Tr(ρlnρ) denotes the von Neumann entropy. Note that the maximum is calculated over the ensembles of separable states $\rho_{k}$ with the probability of occurrence $p_{k}$. The optimal transition rate under infinitely many uses of a channel is given by the classical capacity

(12) $$C\left(\Lambda \right)=\lim_{n\to \infty }\frac{1}{n}\chi \left(\Lambda^{⊗n}\right).$$

In general, C(Λ)≥χ(Λ). However, for a channel Λ with a weakly additive Holevo capacity (χ(Λ⊗Λ)=2χ(Λ)), one has C(Λ)=χ(Λ) [20].

In Reference [41], exact values of the classical capacity were found for certain families of the generalized Pauli channels. Namely, if all $\lambda_{\alpha }\leq 0$ and moreover $\lambda_{1}=\ldots =\lambda_{d}\equiv \lambda_{\max}$, $\lambda_{d+1}=\lambda_{\min}$, then

(13) $$C\left(\Lambda \right)=\frac{1+\left(d-1\right)\lambda_{\min}}{d}\ln\left[1+\left(d-1\right)\lambda_{\min}\right]+\left(d-1\right)\frac{1-\lambda_{\min}}{d}\ln\left(1-\lambda_{\min}\right).$$

In contrast, if all $\lambda_{\alpha }\geq 0$ and also $\lambda_{1}=\lambda_{\max}$, $\lambda_{2}=\ldots =\lambda_{d+1}\equiv \lambda_{\min}$, then

(14) $$C\left(\Lambda \right)=\frac{1+\left(d-1\right)\lambda_{\max}}{d}\ln\left[1+\left(d-1\right)\lambda_{\max}\right]+\left(d-1\right)\frac{1-\lambda_{\max}}{d}\ln\left(1-\lambda_{\max}\right).$$

In addition, if all of the eigenvalues are equal to one another, meaning that $\lambda_{1}=\ldots =\lambda_{d+1}\equiv \lambda$, then one recovers the capacity of the depolarizing channel [42]. For any other combination of eigenvalues, one finds only the lower bound of the classical capacity [41],

(15) $$C_{\mathrm{low}}\left(\Lambda \right)=\max_{\alpha >0}c_{\alpha },\;c_{\alpha }=\frac{1+\left(d-1\right)\lambda_{\alpha }}{d}\ln\left[1+\left(d-1\right)\lambda_{\alpha }\right]+\frac{d-1}{d}\left(1-\lambda_{\alpha }\right)\ln\left(1-\lambda_{\alpha }\right).$$

In the special case of d=2 (the Pauli channels), the above formula gives the exact value of the capacity [21], meaning that $C\left(\Lambda \right)=C_{\mathrm{low}}\left(\Lambda \right)$.

### Generators vs. Memory Kernels

The evolution ρ⟼ρ(t)=Λ(t)[ρ] of an open quantum system is described by a family of time-parameterized quantum channels Λ(t), t≥0, with the initial condition Λ(0)=1l. Such maps can be obtained as solutions to the master equations. In the simplest scenario, the evolution equation $\dot{\Lambda }\left(t\right)=L\Lambda \left(t\right)$, where L is the Gorini–Kossakowski–Sudarshan–Landblad (GKSL) generator [43,44]. The solution to this equation is the Markovian semigroup Λ(t)=exp(tL). For the generalized Pauli channels, one has [26]

(16) $$L=\sum_{\alpha =1}^{d+1}\gamma_{\alpha }L_{\alpha }$$

with the decoherence rates $\gamma_{\alpha }\geq 0$ and

(17) $$L_{\alpha }\left[\rho \right]=\frac{1}{d}\left[\sum_{k=1}^{d-1}U_{\alpha }^{k}\rho U_{\alpha }^{k†}-\left(d-1\right)\rho \right].$$

Generators that are constant in time are sufficient for open system dynamics with a weak coupling to the environment. When this coupling is relatively strong, however, it becomes essential to consider the master equations that take non-Markovian memory effects into account. One generalization of the semigroup master equation is $\dot{\Lambda }\left(t\right)=L\left(t\right)\Lambda \left(t\right)$, where the constant generator is replaced with the time-local generator L(t). In the case of the generalized Pauli channels, one simply has

(18) $$L\left(t\right)=\sum_{\alpha =1}^{d+1}\gamma_{\alpha }\left(t\right)L_{\alpha }.$$

The condition on the decoherence rates is relaxed, as they no longer have to be positive for the dynamics to be legitimate. This time, $\gamma_{\alpha }\left(t\right)\geq 0$ is the necessary and sufficient condition for the corresponding (invertible) Λ(t) to be Markovian in terms of divisibility [45,46]. A dynamical map is CP-divisible if and only if it is decomposable into Λ(t)=V(t,s)Λ(s) for any t≥s≥0. The propagator V(t,s) is then a completely positive, trace-preserving map, and the corresponding evolution is Markovian.

By solving the evolution equation with the time-local generator, we find that the eigenvalues of the associated dynamical map read [26]

(19) $$\lambda_{\alpha }\left(t\right)=\exp\left[\Gamma_{\alpha }\left(t\right)-\Gamma_{0}\left(t\right)\right],$$

where $\Gamma_{\alpha }\left(t\right)=\int_{0}^{t}\gamma_{\alpha }\left(\tau \right)\;d\tau$ for α=0,…,d+1 and $\gamma_{0}\left(t\right)=\sum_{\alpha =1}^{d+1}\gamma_{\alpha }\left(t\right)$. Note that the complete positivity conditions from Equation (10) reduce to

(20) $$\sum_{\alpha =1}^{d+1}e^{\Gamma_{\alpha }\left(t\right)}\leq e^{\Gamma (t)}+d\min_{\beta }e^{\Gamma_{\beta }\left(t\right)}.$$

Another generalization of the Markovian semigroup master equation is realized using memory kernels. In this approach, the GKSL generator is replaced with an integral expression. Now, the evolution of the system is governed by the Nakajima–Zwanzig equation [47,48]

(21) $$\dot{\Lambda }\left(t\right)=\int_{0}^{t}K\left(t-\tau \right)\Lambda \left(\tau \right)\;d\tau ,$$

where K(t) is the memory kernel. Observe that this is an integro-differential equation; therefore, the evolved state ρ(t) depends on every earlier state ρ(τ), τ<t. The memory kernel that corresponds to the generalized Pauli channels has a relatively simple form,

(22) $$K\left(t\right)=\sum_{\alpha =1}^{d+1}k_{\alpha }\left(t\right)L_{\alpha }.$$

Note that K(t) and Λ(t) have common eigenvectors,

(23) $$K\left(t\right)\left[U_{\alpha }^{k}\right]=\kappa_{\alpha }\left(t\right)U_{\alpha }^{k},\;K\left(t\right)\left[I\right]=0,$$

where

(24) $$\kappa_{\alpha }\left(t\right)=k_{\alpha }\left(t\right)-k_{0}\left(t\right)$$

with $k_{0}\left(t\right)=\sum_{\beta =1}^{d+1}k_{\beta }\left(t\right)$ are the eigenvalues of the kernel. Hence, one can rewrite the Nakajima–Zwanzig equation as

(25) $${\dot{\lambda }}_{\alpha }\left(t\right)=\int_{0}^{t}\kappa_{\alpha }\left(t-\tau \right)\lambda_{\alpha }\left(\tau \right)\;d\tau .$$

In the Laplace transform domain, the solution reads

(26) $${\tilde{\lambda }}_{\alpha }\left(s\right)=\frac{1}{s-{\tilde{\kappa }}_{\alpha }\left(s\right)},$$

where $\tilde{f}\left(s\right)=\int_{0}^{\infty }f\left(t\right)e^{-st}dt$ is the Laplace transform of the function f(t).

The necessary and sufficient conditions for legitimate memory kernels are provided in Reference [49]. First, one parameterizes the eigenvalues $\lambda_{\alpha }\left(t\right)$ of the dynamical map by the real function $\ell_{\alpha }\left(t\right)$ in such a way that

(27) $$\lambda_{\alpha }\left(t\right)=1-\int_{0}^{t}\ell_{\alpha }\left(\tau \right)\;d\tau .$$

Now, the associated kernel is legitimate if and only if its eigenvalues

(28) $${\tilde{\kappa }}_{\alpha }\left(s\right)=-\frac{s{\tilde{\ell }}_{\alpha }\left(s\right)}{1-{\tilde{\ell }}_{\alpha }\left(s\right)},$$

where $\ell_{\alpha }\left(t\right)$ satisfies the additional conditions

(29) $$\begin{array}{cc} \int_{0}^{t}\ell_{\alpha }\left(\tau \right)\;d\tau & \geq 0, \end{array}$$

(30) $$\begin{array}{cc} d\int_{0}^{t}\ell_{\alpha }\left(\tau \right)\;d\tau & \leq \sum_{\beta =1}^{d+1}\int_{0}^{t}\ell_{\beta }\left(\tau \right)\;d\tau \leq \frac{d^{2}}{d-1}, \end{array}$$

for α=1,2,…,d+1.

## 4. Engineering Capacity through Kernel Manipulations

In this section, we analyze how the classical capacity of the generalized Pauli channels changes in time for the evolution generated by Equation (21) with the memory kernel

(31) K(t)=δ(t)L+K(t).

Notably, in the formula above, L is a legitimate Markovian semigroup generator from Equation (18) and K(t) is a legitimate, purely non-local memory kernel (i.e., it does not involve the Dirac delta function δ(t)). It is shown that, by adding a non-local part K(t), one can improve the classical capacity of the associated dynamical map Λ(t). The addition of purely local and non-local kernels has already been considered in [9,50], where it was proven that the channel fidelity can be temporarily increased by the appropriate engineering of the kernel parameters. In the following, we consider three types of dynamical maps: the Markovian semigroup $\Lambda^{M}\left(t\right)=e^{tL}$, the non-Markovian noise $\Lambda^{N}\left(t\right)$ that solves ${\dot{\Lambda }}^{N}\left(t\right)=\int_{0}^{t}K\left(t-\tau \right)\Lambda^{N}\left(\tau \right)\;d\tau$, and finally the map Λ(t) that satisfies the Nakajima–Zwanzig equation with K(t)=δ(t)L+K(t). The eigenvalues of the corresponding maps are denoted by $\lambda_{\alpha }^{M}\left(t\right)$, $\lambda_{\alpha }^{N}\left(t\right)$, and $\lambda_{\alpha }\left(t\right)$, respectively. Interestingly, there is no simple relation between the map eigenvalues, as in the Laplace transform domain

(32) $${\tilde{\lambda }}_{\alpha }\left(s\right)=\frac{{\tilde{\lambda }}_{\alpha }^{M}\left(s\right){\tilde{\lambda }}_{\alpha }^{N}\left(s\right)}{{\tilde{\lambda }}_{\alpha }^{M}\left(s\right)+{\tilde{\lambda }}_{\alpha }^{N}\left(s\right)-s{\tilde{\lambda }}_{\alpha }^{M}\left(s\right){\tilde{\lambda }}_{\alpha }^{N}\left(s\right)}.$$

In the following examples, the map that describes the noise part is always non-invertible and not kernel non-decreasing–that is,

(33) $$\exists 0\leq \tau \leq t:\;\ker\Lambda^{N}\left(\tau \right)⊈\ker\Lambda^{N}\left(t\right).$$

In other words, there exists at least one eigenvalue $\lambda_{\alpha }^{N}\left(t\right)$ that reaches zero at some finite time $t_{*}$ but does not remain zero for some $t>t_{*}$. Such dynamical maps are indivisible, and hence the corresponding evolution is non-Markovian [51].

### 4.1. Constant Kernel

First, consider the qubit evolutions (d=2) provided by the isotropic Markovian generator

(34) $$L=\frac{\gamma }{2}\sum_{\alpha =1}^{3}L_{\alpha }$$

with a positive decoherence rate γ and the memory kernel K(t) with constant eigenvalues

(35) $$\kappa_{1}^{N}\left(t\right)=\kappa_{2}^{N}\left(t\right)=-\omega^{2},\;\kappa_{3}^{N}\left(t\right)=0,$$

where ω>0. The corresponding solutions read

(36) $$\lambda_{1}^{M}\left(t\right)=\lambda_{2}^{M}\left(t\right)=\lambda_{3}^{M}\left(t\right)=e^{-\gamma t},$$

and

(37) $$\lambda_{1}^{N}\left(t\right)=\lambda_{2}^{N}\left(t\right)=\cos\omega t,\;\lambda_{3}^{N}\left(t\right)=1,$$

respectively. Observe that the dynamical maps characterized via $\lambda_{\alpha }^{M}\left(t\right)$ and $\lambda_{\alpha }^{N}\left(t\right)$ are always legitimate.

**Figure 1:**
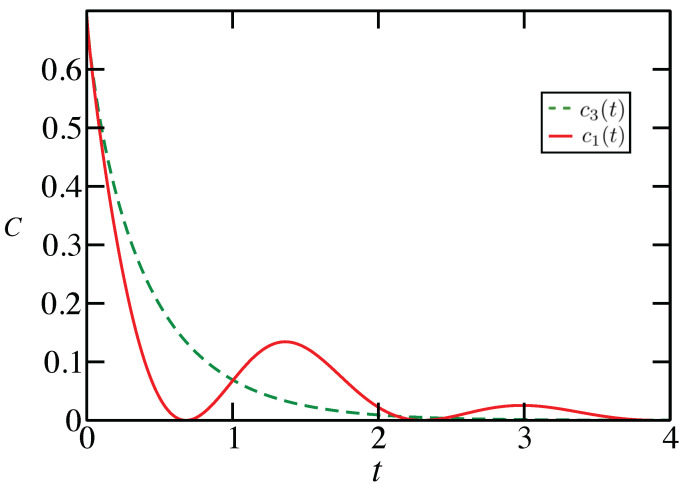
The functions $c_{1}\left(t\right)=c_{2}\left(t\right)$ and $c_{3}\left(t\right)$ are for the qubit evolution with γ=1/s and ω=2/s. The classical capacity of Λ(t) is greater than that of $\Lambda^{M}\left(t\right)$ whenever $c_{1}\left(t\right)>c_{3}\left(t\right)$, or when the solid line lies above the dashed line. The maximal increase in capacity that can be observed for this choice of parameters is around 0.1.

The Pauli dynamical map generated by K(t)=δ(t)L+K(t) is characterized by the following eigenvalues,

(38) $$\lambda_{1}\left(t\right)=\lambda_{2}\left(t\right)=\frac{2\omega }{P}e^{-\gamma t/2}\cos\left(\frac{Pt}{2}+\arctan\frac{\gamma }{P}\right),\;\lambda_{3}\left(t\right)=e^{-\gamma t},$$

where $P=\sqrt{4\omega^{2}-\gamma^{2}}$. The eigenvalues $\lambda_{1}\left(t\right)$ and $\lambda_{2}\left(t\right)$ oscillate if and only if γ<2ω. Additionally, for Λ(t) to describe a legitimate evolution, it is sufficient that

(39) $$\frac{2\omega }{P}\leq \cosh\frac{\gamma t_{*}}{2},$$

where

(40) $$t_{*}=\frac{2}{P}\left(\pi -\arctan\frac{\gamma }{P}\right)$$

is the time corresponding to the first local minimum of the cosine function. This is a direct consequence of the Fujiwara–Algoet conditions from Equation (10). Hence, a combination of two legitimate memory kernels does not necessary yield a physical dynamics. Now, using Equation (15), we can calculate the classical capacity of Λ(t),

(41) $$C\left[\Lambda \left(t\right)\right]=\max\left\{c_{1}\left(t\right),c_{3}\left(t\right)\right\},$$

where $c_{1}\left(t\right)=c_{2}\left(t\right)$ and $c_{3}\left(t\right)=C\left[\Lambda^{M}\left(t\right)\right]$. Therefore, whenever $c_{1}\left(t\right)>c_{3}\left(t\right)$, one observes an increase in capacity for the system with additional noise. An exemplary choice of parameters is shown in Figure 1.

### 4.2. Exponential Decay

Let us take the Markovian semigroup generated by

(42) $$L=\frac{\gamma }{d}\sum_{\alpha =1}^{d+1}L_{\alpha }$$

and the exponentially decaying memory kernel K(t), similar to the one analyzed in [9,50], with

(43) $$\kappa_{\alpha }^{N}\left(t\right)=-\omega^{2}e^{-Zt};\;\kappa_{\alpha_{*}}^{N}\left(t\right)=0,\;\alpha \neq \alpha_{*}.$$

Assume that the constants γ, *Z*, and ω are positive. By solving the master equations, one can find the associated dynamical maps $\Lambda^{M}\left(t\right)$ and $\Lambda^{N}\left(t\right)$, whose eigenvalues are given by

(44) $$\lambda_{\alpha }^{M}\left(t\right)=e^{-\gamma t}$$

and $\lambda_{\alpha_{*}}^{N}\left(t\right)=1$,

(45) $$\lambda_{\alpha }^{N}\left(t\right)=\frac{2\omega }{P}e^{-Zt/2}\cos\left(\frac{Pt}{2}-\arctan\frac{Z}{P}\right)$$

for $\alpha \neq \alpha_{*}$, where $P=\sqrt{4\omega^{2}-Z^{2}}$. Note that for Z=γ, Equation (45) is very similar to $\lambda_{1}\left(t\right)$ from Equation (38) but differs in the sign before the arcus tangent. The map $\Lambda^{M}\left(t\right)$ is always legitimate, whereas $\Lambda^{N}\left(t\right)$ describes a physical dynamics if

(46) $$e^{Zt_{*}/2}\geq \frac{2(d-1)\omega }{P},$$

where

(47) $$t_{*}=\frac{2}{P}\left(\pi +\arctan\frac{Z}{P}\right)$$

corresponds to the first local minimum of the cosine function.

Now, we analyze the behavior of the dynamical map obtained using K(t)=δ(t)L+K(t). Namely, after adding the non-Markovian noise to the semigroup, the eigenvalue $\lambda_{\alpha_{*}}\left(t\right)=e^{-\gamma t}$ remains unchanged. On the other hand,

(48) $$\lambda_{\alpha }\left(t\right)=\frac{2\omega }{R}e^{-(\gamma +Z)t/2}\cos\left(\frac{Rt}{2}+\arctan\frac{\gamma -Z}{R}\right),$$

for $\alpha \neq \alpha_{*}$, where $R=\sqrt{4\omega^{2}-{\left(\gamma -Z\right)}^{2}}$. Note that Equation (48) is not a simple shift of Equation (45) by Z⟼γ−Z, as there are two additional sign differences. For d=2, a sufficient condition for Λ(t) to produce a legitimate evolution is

(49) $$\frac{2\omega }{R}\leq e^{Zt_{*}/2}\cosh\frac{\gamma t_{*}}{2},$$

where this time

(50) $$t_{*}=\frac{2}{R}\left(\pi -\arctan\frac{\gamma -Z}{R}\right).$$

Unfortunately, the complete positivity conditions for d≥3 cannot be simplified in a similar manner. Assuming that Λ(t) describes a qudit evolution, Equation (15) gives the following formula for the lower bound of the classical capacity of Λ(t),

(51) $$C\left[\Lambda \left(t\right)\right]=\max\left\{c_{\alpha }\left(t\right),c_{\alpha_{*}}\left(t\right)\right\}.$$

**Figure 2:**
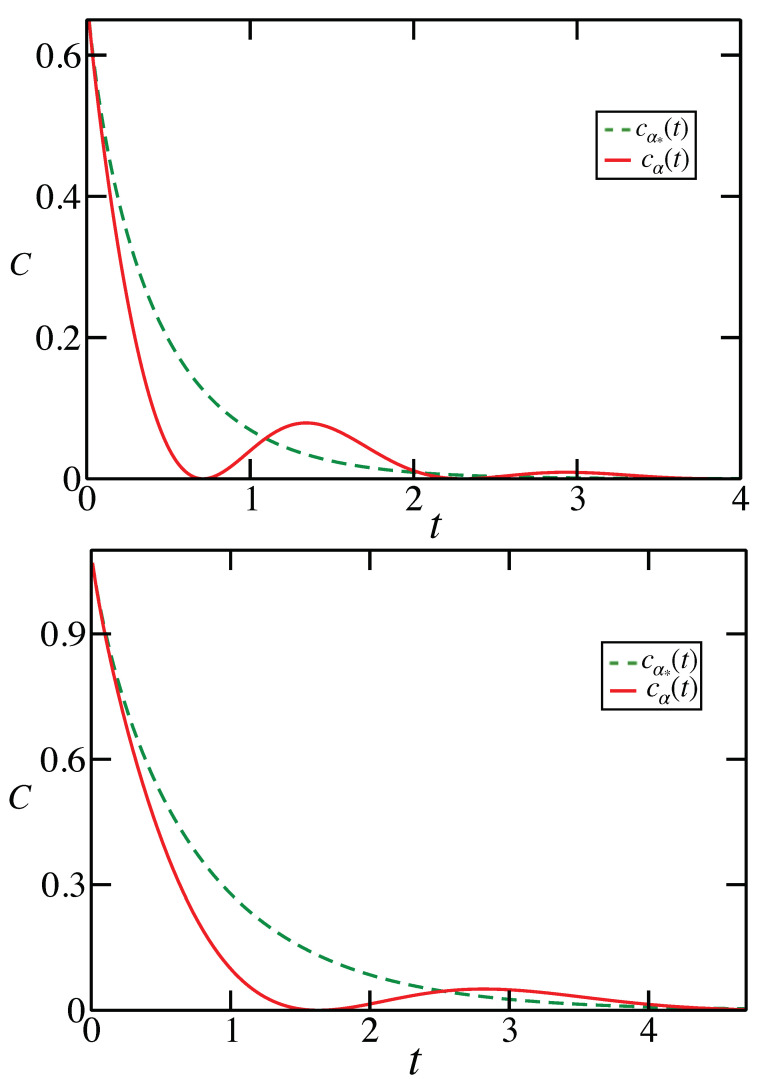
For the functions $c_{\alpha_{*}}\left(t\right)$ and $c_{\alpha }\left(t\right)$, $\alpha \neq \alpha_{*}$ for the qubit evolution with γ=1/s, Z=1/(3s), and ω=2/s (**top**), as well as for the qutrit evolution with γ=3/(5s), Z=1/(5s), and ω=9/(10s) (**bottom**). The introduction of noise into the Markovian evolution results in an increased classical capacity for the time intervals in which $c_{\alpha }\left(t\right)>c_{\alpha_{*}}\left(t\right)$. This corresponds to the situations when the solid line is above the dashed line. A greater enhancement is observed for the lower-dimensional system.

Observe that $c_{\alpha_{*}}\left(t\right)=C\left[\Lambda^{M}\left(t\right)\right]$; hence, the channel capacity for Λ(t) is greater than for the Markovian evolution if $c_{\alpha }\left(t\right)>c_{\alpha_{*}}\left(t\right)$. Two examples of appropriate parameter engineering are presented in Figure 2.

### 4.3. Beyond the Semigroup

The classical capacity can also be enhanced in a more general case. Let us consider the Markovian evolution characterized by a dynamical map $\Lambda^{M}\left(t\right)$ that is not a semigroup. Instead, it is generated via the time-local generator $L^{M}\left(t\right)$ from Equation (18) with $\gamma_{\alpha }^{M}\left(t\right)\geq 0$. Now, the most natural way to introduce noise is to add the generator $L^{N}\left(t\right)$ of a non-Markovian evolution, where at least one decoherence rate $\gamma_{\alpha }^{N}\left(t\right)≱0$. The resulting dynamical map Λ(t) is provided via

(52) $$L\left(t\right)=L^{M}\left(t\right)+L^{N}\left(t\right).$$

From a physical point of view, one can add two legitimate generators when the environmental cross-correlations can be ignored [52]. Now, the eigenvalues of the generalized Pauli map Λ(t) read

(53) $$\lambda_{\alpha }\left(t\right)=\lambda_{\alpha }^{M}\left(t\right)\lambda_{\alpha }^{N}\left(t\right),$$

which means that $\Lambda \left(t\right)=\Lambda^{M}\left(t\right)\Lambda^{N}\left(t\right)$ is a composition of two (commutative) generalized Pauli dynamical maps. However, due to the fact that $\lambda_{\alpha }\left(t\right)\geq 0$ for any Λ(t) that arises from a legitimate time-local generator, $\lambda_{\alpha }\left(t\right)\leq \lambda_{\alpha }^{M}\left(t\right)$. Therefore, there can be no increase in the classical capacity. Hence, let us instead consider a more general form of the memory kernel K(t). Namely, we can replace the semigroup generator L in Equation (31) with the memory kernel K(t) that describes the same evolution as the time-local generator L(t). Then, one has

(54) K(t)=K(t)+K(t),

where K(t) and K(t) correspond to a Markovian and non-Markovian dynamics, respectively.

As a case study, we analyze the evolution where the Markovian part is given by the generator

(55) $$L^{M}\left(t\right)=\frac{r}{d+e^{rt}}\sum_{\alpha =1}^{d+1}L_{\alpha }$$

with r>0. The solution reads

(56) $$\lambda_{\alpha }^{M}\left(t\right)=\frac{1+de^{-rt}}{d+1},$$

and $\Lambda^{M}\left(t\right)$ is always completely positive. One finds that the corresponding kernel has the eigenvalues

(57) $$\kappa_{\alpha }^{M}\left(t\right)=-\frac{dr}{d+1}\left(\delta \left(t\right)-\frac{r}{d+1}e^{-\frac{rt}{d+1}}\right).$$

Therefore, from the kernel point of view, our generalization means that the Markovian part of the kernel not only has terms proportional to the Dirac delta but also has some purely non-local parts. The environmental noise is realized with $\kappa_{\alpha }^{N}\left(t\right)$ from Equation (43) for a fixed $Z=\frac{r}{d+1}$. The associated solution is $\lambda_{\alpha_{*}}^{N}\left(t\right)=1$ and

(58) $$\lambda_{\alpha }^{N}\left(t\right)=\frac{2\omega }{P}e^{-\frac{rt}{2(d+1)}}\cos\left(\frac{Pt}{2}-\arctan\frac{r}{P(d+1)}\right)$$

for $\alpha \neq \alpha_{*}$, where $P=\sqrt{4\omega^{2}-r^{2}/{\left(d+1\right)}^{2}}$. For the complete positivity condition, see Equation (46). Finally, the dynamical map generated by K(t)=K(t)+K(t) is characterized by $\lambda_{\alpha_{*}}\left(t\right)=\lambda_{\alpha }^{M}\left(t\right)$ and

(59) $$\lambda_{\alpha }\left(t\right)=\frac{2X}{(d+1)Y}e^{-\frac{rt}{2}}\cos\left(\frac{Yt}{2}+\arctan\frac{r(d-1)}{Y(d+1)}\right),$$

where $\alpha \neq \alpha_{*}$, $Y=\sqrt{4\omega^{2}-r^{2}}$, and $X=\sqrt{{\left(d+1\right)}^{2}\omega^{2}-dr^{2}}$. For this map to describe a physical evolution in d=2 and d=3, it is sufficient that

(60) $$\frac{X}{Y}\leq \frac{1}{d-1}e^{rt/2}+\frac{1}{2}e^{-rt/2}$$

with the first minimum of the cosine function corresponding to

(61) $$t_{*}=\frac{2}{Y}\left(\pi -\arctan\frac{(d-1)r}{(d+1)Y}\right).$$

**Figure 3:**
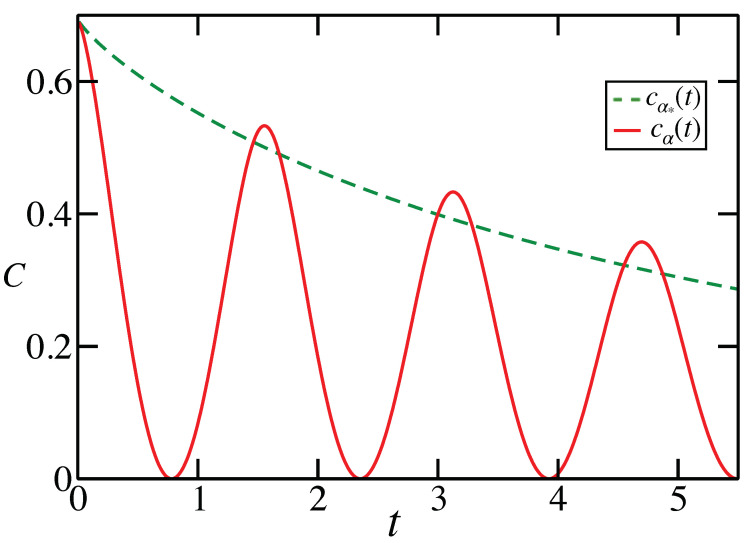
For the functions $c_{\alpha_{*}}\left(t\right)$ and $c_{\alpha }\left(t\right)$, $\alpha \neq \alpha_{*}$, for the qubit evolution with r=1/(10s), and ω=2/s. Observe that $C\left[\Lambda \left(t\right)\right]>C\left[\Lambda^{M}\left(t\right)\right]$ when $c_{\alpha }\left(t\right)>c_{\alpha_{*}}\left(t\right)$, or, in other words, when the dashed line lies below the solid line. Contrary to the semigroup examples, the maximal capacity increase does not occur during the first time range when the classical capacity is enhanced.

Analogically to the previous example, the lower bound for the classical capacity of Λ(t) is given by

(62) $$C\left[\Lambda \left(t\right)\right]=\max\left\{c_{\alpha }\left(t\right),c_{\alpha_{*}}\left(t\right)\right\},$$

for $c_{\alpha }\left(t\right)$ defined in Equation (15), where $C\left[\Lambda^{M}\left(t\right)\right]=c_{\alpha_{*}}\left(t\right)$ is the capacity of the Markovian evolution. Again, we observe a temporary increase in the channel capacity for a certain set of kernel parameters (see Figure 3 for the qubit evolution).

## 5. Conclusions

We analyzed the classical capacity of generalized Pauli channels generated via memory kernel master equations. We compared the evolution of channel capacity for the Markovian semigroup and for the dynamical map generated via a memory kernel that is a sum of the Markovian part and the noise part. Note that the local part is legitimate and identical for both maps. The non-local part, which corresponds to environmental noise, was chosen in such a way that the dynamical map that solves the associated Nakajima–Zwanzig equation describes a valid physical evolution. It was found that the introduction of noise into the master equation could lead to a temporary increase in the classical capacity. In other words, noise effects can be beneficial in quantum information processing, as they result in the enhanced ability of a quantum channel to reliably transmit classical information. Similar results were obtained after a generalization of the Markovian semigroup to a Markovian evolution provided by a time-local generator. However, we showed that analogical observations cannot be made for time-local master equations. A dynamical map generated via the sum of two time-local generators never produces a classical capacity that is higher than that of a map that arises from a single generator.

It would be interesting to further analyze this topic by considering the kernels for noninvertible Markovian dynamical maps mixed with the noise kernels. Another open question concerns the relation between quantum maps that increase classical capacity and maps that increase the channel fidelity. One could expect that capacity enhancement means higher fidelity, but not the other way around. A comparative analysis could also be performed for other important measures, such as output purity, concurrence, logarithmic negativity, and von Neumann entropy.

## Data Availability

Not applicable.
